# Adaptive coping strategies in patients with chronic pain conditions and their interpretation of disease

**DOI:** 10.1186/1471-2458-10-507

**Published:** 2010-08-20

**Authors:** Arndt Büssing, Thomas Ostermann, Edmund AM Neugebauer, Peter Heusser

**Affiliations:** 1 Center for Integrative Medicine, University Witten/Herdecke (Germany), Gerhard-Kienle-Weg 4, 58313 Herdecke, Germany; 2 Institute for Research in Operative Medicine, Chair for Surgical Research, University of Witten/Herdecke, Witten, Germany

## Abstract

**Background:**

We examined which adaptive coping strategies, referring to the concept of 'locus of disease control', were of relevance for patients with chronic pain conditions, and how they were interconnected with patients' life satisfaction and interpretation of disease.

**Methods:**

In a multicenter cross-sectional anonymous survey with the AKU questionnaire, we enrolled 579 patients (mean age 54 ± 14 years) with various chronic pain conditions.

**Results:**

Disease as an adverse interruption of life was the prevalent interpretation of chronic pain conditions. As a consequence, patients relied on external powerful sources to control their disease (i.e., *Trust in Medical Help*; *Search for Information and Alternative Help*), but also on internal powers and virtues (i.e., *Conscious Way of Living*; *Positive Attitudes*). In contrast, *Trust in Divine Help *as an external transcendent source and *Reappraisal: Illness as Chance *as an internal (cognitive) strategy were valued moderately. Regression analyses indicated that *Positive Attitudes *and higher age were significant predictors of patients' life satisfaction, but none of the other adaptive coping strategies. While the adaptive coping strategies were not associated with negative interpretations of disease, the cognitive reappraisal attitude was of significant relevance for positive interpretations such as value and challenge.

**Conclusions:**

The experience of illness may enhance intensity and depth of life, and thus one may explain the association between internal adaptive coping strategies (particularly *Reappraisal*) and positive interpretations of disease. To restore a sense of self-control over pain (and thus congruence with the situation), and the conviction that one is not necessarily disabled by disease, is a major task in patient care. In the context of health services research, apart from effective pain management, a comprehensive approach is needed which enhances the psycho-spiritual well-being of patients.

## Background

There are different ways to cope with pain, and there are different ways to regulate emotions associated with chronic diseases. Because most patients with chronic diseases are unable to 'solve' their persisting pain conditions by themselves (in terms of recovery or repair) and to find distance to negative emotions associated with pain, they have to find strategies to adapt to a long-lasting course of disease. Patients have to find ways to maintain physical, emotional and spiritual health despite of often long-lasting courses. Thus, patients' coping with chronic pain is an ongoing process which includes appraisals of stress, cognitive, behavioural, and emotional coping responses, and subsequent reappraisals of stress.

One of the most frequently used concept on adaptation strategies of patients with chronic pain diseases differentiates active and passive coping [1,2]. Active coping (i.e., problem solving, including collecting information and refocusing on the problem, or regulation of emotion by focusing attention on the emotional response aroused by the stressor) is associated with less pain, less depression, less functional impairment, and higher general self-efficacy, while passive coping (i.e., avoidance and escape) is correlated with reports of greater depression, greater pain and flare-up activity, greater functional impairment, and lower general self-efficacy [1]. Although the importance of decreasing maladaptive and encouraging adaptive coping responses is emphasized by innovative treatment programs for chronic pain, one nevertheless has to ask which adaptive coping strategies were of relevance for the patients.

A recent meta-analysis found that among older adults with persistent pain, the most frequently reported coping strategies were Task Persistence (maintaining activity, for example despite fluctuations of pain intensity), Pacing (activity avoidance), and Coping Self-Statements (a form of conditioning to put a stop for example to thoughts that lead to anxiety etc. and to replace them with rational thoughts); the least frequently used strategies were Asking for Assistance and Relaxation [3]. Findings from that study suggest useful coping strategies clinicians could explore with individual patients [3].

Our own study results suggest that most patients with chronic diseases use adaptive coping strategies which can be differentiated according to the utilization of external resources of health control (i.e., *Trust in Medical Help*; *Search for Information and Alternative Help; Trust in Divine Help*) and internal sources (i.e., *Conscious Way of Living*; *Positive Attitudes*; *Reappraisal: Illness as Chance*) [4,5]. Particular the cognitive reappraisal strategy was of outstanding relevance. It deals with patients' interpretation of disease as an opportunity, a hint to change life, and reflect upon what is essential in life. Because of this reflection, patients may alter their goals, change aspects of life or behaviour, and may see their situation as a chance for personal growth (transformation). However, the subjective meaning of illness is influenced by intrapersonal, disease-related and environmental factors [6]. These interpretations of illness may have an influence on preferences in decision-making and choice of coping strategies.

In this report, we intended to analyze which adaptive coping strategies referring to the concept of 'locus of health control' (in terms of external or internal resources), were of relevance for patients with chronic pain conditions, and how these strategies were associated with patients' life satisfaction and interpretation of illness. Our hypothesis was that particularly the adaptive coping strategies referring on the internal resources are associated with life satisfaction, while positive interpretations of illness (such as challenge or value) are related to reappraisal processes.

## Methods

### Patients

For this multicenter cross-sectional survey, patients were recruited from the acute pain outpatient clinic of the Communal Hospital in Herdecke, from the Department of Internal and Integrative Medicine at the Essen-Mitte Clinics, from the Orthopaedic Clinic in Bad Bocklet and from the orthopaedic Baumrain Clinic in Bad Berleburg. Their institutional heads gave approval to run this anonymous survey. All enrolled individuals were informed of the purpose of the study, were assured of confidentiality, and consented to participate. The questionnaires were anonymous (and asked neither for names, addresses or clinical details - with the exception of a diagnosis), and the pooled data could not be tracked back to individual patients.

To minimize the bias of a 'convenience sample', different medical centres in West-Germany were chosen, and patients were recruited consecutively as they attended the respective clinics. To obtain a more naturalistic sample, we had neither inclusion nor exclusion criteria (with the exception of the diagnosis chronic pain disease and consent to participate). We did not measure pain intensity scores, and thus we categorized the patients according to the recruiting source which indicated differences with respect to the need for acute interventions (which is given in the out-patient clinic offering predominantly acute pain relieving interventions, as contrasted to rehabilitation clinics which offer predominantly orthopaedic interventions and medication, and the internal and integrative medicine clinic offering mind-body programs, naturopathy and medication).

The demographic data of 579 (out of 607) patients which provided enough data for statistical analyses were depicted in table 1. The underlying pain diseases were heterogeneous: 15% had spine-associated pain syndromes (low back pain etc.), 12% fibromyalgia, 8% polyarthritis/-arthrosis, 4% migraine/headache, 4% chronic inflammatory bowel diseases, 4% cancer (accompanied by pain), 8% amputations accompanied by pain, 4% pain associated with psycho-physical exhaustion, and 40.5% various other or unclear pain diagnoses (i.e., "pain syndrome", "general pain", etc.) categorized as "others". In most cases, the chronic pain conditions were not associated with work injuries or post surgical conditions. However, one recruiting centre added exclusively patients with phantom pain after limb amputations.

**Table 1:** Demographic data of patients with chronic pain conditions

	All patients
**Gender (%)**	
women	77
men	23

**Age **(years)	54.3 ± 14.4

**Family status **(%)	
married	48
living with partner	10
divorced	14
living alone	15
widowed	14

**Educational level **(%)	
secondary (Hauptschule)	48
junior high school (Realschule)	23
high school (Gymnasium)	15
other	13

**Religious denomination **(%)	
christian	83
others	4
none	13

**Underlying pain conditions **(%)	
spine-associated pain syndromes	15
fibromyalgia	12
polyarthritis/-arthrosis	8
migraine/headache	4
chronic inflammatory bowel diseases	4
cancer (accompanied by pain)	4
amputations (accompanied by pain)	8
pain associated with psycho-physical exhaustion	4
other pain syndromes or diseases	41

**Duration of disease **(months)	96 ± 116

**Life Satisfaction **(% Score)	67 ± 18

**Escape from Illness **(% Score)	52 ± 27

## Measures

Adaptive coping strategies in response to chronic pain conditions were measured with the AKU questionnaire (AKU is an acronym of the German translation of "Adaptive Coping with Disease"), which was designed to identify adaptive coping styles, such as to create favourable conditions, search for information, medical support, religious support, social support, initiative spirit, and positive (re)interpretation of disease [4,5,7]. The underlying concept of the instrument refers to internal and external loci of disease/health control based on the work of Rotter [8,9] and Levenson [10]. The questionnaire was re-validated recently in a sample of 6,963 individuals, and we were able to approve the 6 factorial structure of the 28-item instrument which had a good internal consistency (Cronbach's alpha = 0.867; difficulty index 0.67) [5], i.e.:

▪ *Trust in Divine Help *in response to disease addresses non-organized intrinsic religiosity as an external transcendent resource to cope (i.e., trust in a higher power which carries through; strong belief that God will help; faith is a strong hold, even in hard times; pray to become healthy again; live in accordance with religious convictions).

▪ *Trust in Medial Help *addresses patients' reliance on an external medical source of health control (i.e., trust in the therapeutic potentials of modern medicine, take prescribed medicaments, follow advises of medicals, full confidence in doctors and therapists).

▪ *Search for Information and Alternative Help *refers to external sources providing additional information or alternative help (i.e., thoroughly informed about disease; get thorough information how to become healthy again; find people which can help; search for alternative ways of healing).

▪ *Conscious Way of Living *addresses cognitive and behavioural strategies in terms of internal powers and virtues (i.e., healthy diet; physical fitness; living consciously; keep away harmful influences; change life to get well).

▪ *Positive Attitudes *refers to internal cognitive and behavioural strategies (i.e., realization of shelved dreams and wishes; resolving cumbering situations of the past; take life in own hands; doing all that what pleases; positive thinking; avoiding thinking at illness).

▪ *Reappraisal: Illness as Chance *addresses a reappraisal attitude referring to cognitive processes of life reflection (i.e., reflect on what is essential in life; illness has meaning; illness as a chance for development; appreciation of life because of illness).

The items of the AKU were scored on a 5-point scale from disagreement to agreement (0 - does not apply at all; 1 - does not truly apply; 2 - don't know; 3 - applies quite a bit; 4 - applies very much). The sum scores were referred to a 100% level (transformed scale score). Scores > 50% indicate high agreement or utilization of coping strategy, while scores < 50% indicate low usage of respective strategy.

The questionnaire holds 3 independent items, which did not contribute to the primary AKU item pool. They made up an independent scale termed *Escape from illness *(i.e., fear what illness will bring; would like to run away from illness; when I wake up, I don't know how to face the day", which addresses a passive (avoidance-escape) coping style [4,5,7], while the AKU questionnaire differentiates active adaptive coping styles. It was confirmed recently that *Escape *correlated strongly with depression, with disease appraisals such as 'weakness/failure' and 'punishment', and negatively with life satisfaction [11]. The items were scored on a 5-point scale from disagreement to agreement.

To measure how the patients interpret their disease, we used the 'Interpretation of Illness Questionnaire' (IIQ) [12] which refers to the work of the Canadian psychiatrist Lipowski [13]. The 8-item instrument has satisfactory internal consistency (Cronbach's alpha = 0.730) and involves guilt-associated negative interpretations (i.e., punishment, weakness), fatalistic negative interpretations (i.e., adverse interruption of life/loss, threat/enemy), strategy-associated interpretations (i.e., relieving break from the demands of life, call for help), and positive interpretations of disease (i.e., challenge, value) [12]. The items were scored on a 5-point scale from disagreement to agreement (0 - does not apply at all; 1 - does not truly apply; 2 - don't know; 3 - applies quite a bit; 4 - applies very much), and are referred to a 100% level (4 "regularly" = 100%).

Life satisfaction was measured with the Brief Multidimensional Life Satisfaction Scale (BMLSS) [14]. The eight items of the BMLSS refer to intrinsic dimensions (Myself, Overall Life), social dimensions (Friendships, Family life), external dimension (Work, Where I live), and the perspective dimension (Financial Situation, Future Prospects). All items were scored on a 7-point scale from dissatisfaction to satisfaction (0 - Terrible; 1 - Unhappy; 2 - Mostly dissatisfied; 3 - Mixed (about equally satisfied and dissatisfied); 4 - Mostly satisfied; 5 - Pleased; 6 - Delighted). The Life Satisfaction sum score was referred to a 100% level (transformed scale score). Scores > 50% indicate high life satisfaction, while scores < 50% indicate low satisfaction.

### Statistical analysis

Analyses of variance (ANOVA), correlation and stepwise regression analyses were performed with SPSS for Windows 17.0. We judged p < 0.01 as significant. With respect to the correlation analyses, r > .5 is regarded as a strong correlation, r between .3 and .5 as a moderate correlation, while r between .2 and .3 is regarded as a weak correlation, and r < .2 as no or negligible correlation.

## Results

### Demographic characteristics of patients

We analyzed data of 579 patients (mean age 54 ± 14 years) with chronic pain conditions (mean duration of disease: 96 ± 116 months). As shown in Table 1, most patients were living with a partner, had a lower educational level, and a Christian denomination. We had a predominance of female patients (77%), which is in line with findings of Munce and Steward [15], who reported that women had higher rates of chronic pain conditions and depression than men. However, *Escape from illness*, as a passive avoidance-escape strategy, was not a major issue to the patients (Table 1); instead, Life Satisfaction scores were moderately expressed, indicating that the patients were mostly satisfied.

### Adaptive coping in patients with chronic pain conditions

The patients with chronic pain conditions analyzed herein relied on both external powerful sources to control their disease (i.e., *Trust in Medical Help*; *Search for Information and Alternative Help*) and on internal powers and virtues (i.e., *Conscious and Healthy Way of Living*, *Positive Attitudes*), while the transcendent external locus of disease control (i.e., *Trust in Divine Help*), and also *Reappraisal: Illness as Chance *were valued moderately (Table 2). With respect to age, underlying pain conditions and burden of pain (as an indirect measure we investigated which clinic was seen for treatment, i.e., the acute pain outpatient clinic offers predominantly acute pharmaceutical interventions, while the rehabilitation clinics offers predominantly orthopaedic interventions and medication, as contrasted by mind-body programs, naturopathy and medication in the internal and integrative medicine clinic) we found several significant differences which are depicted in Table 2. The utilization of the respective adaptive coping strategies did not significantly differ with respect to gender (Table 2), while the educational level had a small impact on *Trust in Medical Help*, which was the highest in patients with low educational level (F = 3.2; p = 0.022). Age had a significant (p < .0001) impact on *Trust in Divine Help *(F = 10.4), *Trust in Medical Help *(F = 5.2) and *Conscious Way of Living *(F = 4.8). Duration of disease had no significant impact on the adaptive coping strategies (F < 2.0; n.s.); however, *Conscious Way of Living *showed in trend some degree of variance (F = 2.3; p = .053).

**Table 2:** Adaptive coping styles in patients with chronic pain diseases

		TDH	TMH	SIAH	PoA	CWoL	RIC	Escape
		*External locus of control*			*Internal locus of control*			/

**all**	Mean	56.34	79.28	75.16	70.64	72.16	50.43	52.00
	SD	32.56	20.18	21.07	16.75	17.17	26.33	27.04

**Gender**								
women	Mean	57.60	79.03	75.65	71.20	72.56	51.11	51.89
	SD	32.20	20.70	21.52	16.76	17.03	26.67	26.91
men	Mean	51.94	80.15	73.42	68.65	70.77	48.09	49.12
	SD	33.52	18.33	19.44	16.64	17.65	25.06	26.17
F-value		2.834	.291	1.067	2.240	1.046	1.199	1.614
p-value		0.093	n.s.	n.s.	n.s.	n.s.	n.s.	n.s.

**age**								
<30 years	Mean	**41.03**	72.06	67.03	69.26	65.88	51.96	52.40
	SD	35.03	18.18	18.09	13.62	17.64	25.98	18.14
31-40 years	Mean	**41.94**	79.17	75.22	66.94	65.81	48.97	51.89
	SD	30.59	14.19	20.04	16.67	19.59	26.57	26.89
41-50 years	Mean	50.60	73.97	76.02	68.91	70.32	48.70	45.97
	SD	33.05	20.81	16.82	18.37	16.68	26.81	27.37
51-60 years	Mean	57.82	81.77	76.59	71.35	73.97	51.12	54.64
	SD	29.65	16.39	20.85	15.33	15.20	25.71	26.92
61-70 years	Mean	63.77	85.34	75.06	71.98	74.28	52.40	54.46
	SD	31.35	20.16	23.51	18.09	17.87	26.59	27.65
>70 years	Mean	**73.22**	79.05	74.97	74.03	76.73	49.35	53.36
	SD	29.66	26.93	26.01	15.41	15.98	27.03	27.93
F-value		**10.356**	**5.208**	1.196	1.764	**4.844**	0.319	1.755
p-value		**<0.0001**	**<0.0001**	n.s.	n.s.	**0.002**	n.s.	n.s.

**Underlying disease**								
spine-associated pain syndromes	Mean	57.08	80.06	74.65	67.69	71.16	47.52	54.81
	SD	31.78	19.02	21.00	17.41	17.67	27.64	21.88
fibromyalgia	Mean	57.40	80.18	76.77	67.92	73.41	50.22	56.08
	SD	29.09	18.70	18.48	16.22	13.12	26.17	27.44
polyarthritis/-arthrosis	Mean	59.91	81.67	81.39	73.06	75.36	48.86	50.95
	SD	27.73	19.96	16.43	14.30	13.67	23.82	27.25
migraine/headache	Mean	50.42	77.85	73.07	68.40	69.40	44.83	57.54
	SD	32.38	20.45	24.62	15.10	21.63	30.98	30.15
chronic inflammatory bowel diseases	Mean	**33.60**	71.92	69.53	65.97	65.13	53.08	50.05
	SD	37.65	21.71	26.79	13.90	19.30	20.13	27.63
cancer (accompanied by pain)	Mean	57.74	73.21	77.08	71.03	72.86	50.60	51.09
	SD	30.72	27.68	18.78	13.08	17.51	26.90	26.39
amputations (accompanied by pain)	Mean	**69.77**	89.53	76.63	78.95	81.03	57.97	46.00
	SD	29.49	18.17	22.54	15.22	15.23	29.38	27.50
pain associated with psycho-physical exhaustion	Mean	51.35	71.39	69.47	69.58	72.12	55.93	45.74
	SD	32.39	17.06	23.14	16.45	18.23	21.47	27.95
other pain syndromes	Mean	56.63	78.60	74.40	71.60	71.34	50.73	52.78
	SD	33.50	20.54	21.73	17.45	17.16	26.11	26.77
F-value		**2.649**	2.490	1.082	2.398	2.354	0.816	0.894
p-value		**0.007**	0.012	n.s.	0.015	0.017	n.s.	n.s.

**Pain treatment**								
Pain outpatient Clinic	Mean	61.82	86.96	78.37	71.21	73.25	54.36	**60.31**
	SD	31.73	13.94	18.71	17.70	16.49	26.54	25.50
Rehabilitation Clinic	Mean	60.76	82.77	71.45	70.19	75.64	50.78	55.97
	SD	29.27	18.83	22.12	17.39	15.35	25.66	27.49
Mind-Body/CAM Training Programs	Mean	51.94	73.84	74.71	70.50	70.50	48.49	46.31
	SD	33.35	21.96	21.97	15.87	17.61	26.13	26.35
F-value		**6.048**	**25.536**	3.568	0.143	4.283	2.474	**15.674**
p-value		**0.003**	**<0.0001**	0.029	n.s.	0.014	0.085	**<0.0001**

Scores > 50% represent a positive attitude (agreement), while scores < 50% represent a negative attitude (disagreement). Deviations > 15% from the mean were highlighted

*Abbreviations: *TDH - Trust in Divine Help; TMH - Trust in Medical Help; SIAH - Search for Information/Alternative Help; PoA - Positive Attitudes; CWoL - Conscious Way of Living; RIC - Reappraisal: Illness as Chance; Esc -Escape from Illness

It was obvious that patients from the acute outpatient clinic had significantly higher scores for *Trust in Medical Help *and *Escape from Illness *than patients from the rehabilitation clinic or patients attending the mind-body program, and were also in *Search for Information and Alternative Help*. This may indicate higher need for external help.

### Interpretation of disease in patients with chronic pain conditions

**Figure 1:**
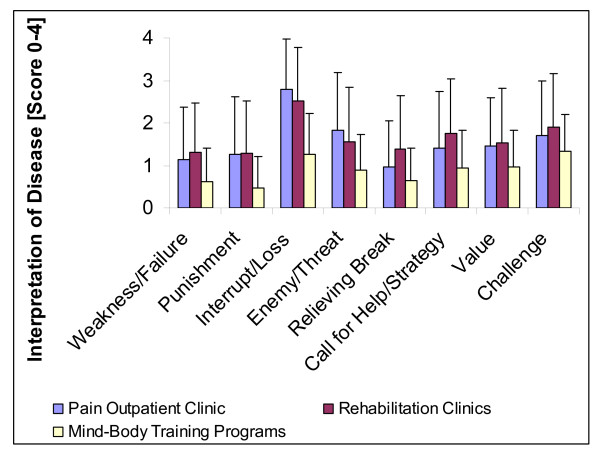
**Interpretation of Illness in patients with chronic pain diseases**.

Most patients regarded their disease as an adverse Interruption (Loss) of life (Figure 1), particularly patients attending the acute pain outpatient clinic. Guilt-associated negative interpretations (i.e., Punishment, Weakness) were rejected in most cases, while positive interpretations of disease (i.e., Challenge, Value) were of some relevance. However, in all cases the patients from the clinic offering Mind-Body/naturopathy intervention programs had significantly lower scores as compared to the patients from the orthopaedic rehabilitation clinics and the acute pain outpatient clinic (F between 13.2 and 117.5; p < 0.0001).

### Associations between adaptive coping strategies, life satisfaction and interpretation of disease

To analyse how the adaptive coping strategies, life satisfaction and interpretation of disease were associated, we first performed correlation analyses (Table 3).

**Table 3:** Correlations between Adaptive Coping Styles and other dimensions

	TDH	TMH	SIAH	PoA	CWoL	RIC	Escape
	*External locus of control*			*Internal locus of control*			/

**Adaptive coping strategies**							
Trust in Divine Help	1.000	.238**	.281**	.261**	.344**	.417**	-.017
Trust in Medical Help		1.000	.389**	.222**	.247**	.060	.082
Search Information/Alternative Help			1.000	.402**	.387**	.272**	-.068
Positive Attitudes				1.000	.542**	.280**	-.218 **
Conscious Way of Living					1.000	.259**	-.093
Reappraisal: Illness as Chance						1.000	-.009

**Life Satisfaction**	.172*	.149	.176*	.393**	.361**	.081	-.227 **

**Interpretation of Disease**							
Weakness/Failure	.057	-.001	-.100	-.102	-.096	.160**	.272**
Punishment	.011	.027	-.108	-.091	-.083	.074	.373**
Loss/Interruption	.015	.180**	.039	-.067	-.017	-.033	.394**
Enemy/threat	.006	.021	-.036	-.086	-.059	.034	.441**
Relieving break	.090	.006	-.096	-.003	-.007	.215**	.079
Call for help/Strategy	.131*	-.021	-.024	-.079	-.044	.229**	.171**
Value	.290**	.030	.135*	.171**	.156**	.469**	-.072
Challenge	.223**	.066	.132*	.251**	.188**	.408**	-.101

** p < .0001; *p < .01 (Spearman-Rho, 2-tailed)

*Abbreviations: *TDH - Trust in Divine Help; TMH - Trust in Medical Help; SIAH - Search for Information/Alternative Help; PoA - Positive Attitudes; CWoL - Conscious Way of Living; RIC - Reappraisal: Illness as Chance; Esc -Escape from Illness

Among the intrinsic styles, *Conscious and Healthy Way of Living *correlated strongly with *Positive Attitudes*, and moderately with *Search for Information/Alternative Help *and *Trust in Devine Help*, while *Positive Attitudes *were just weakly associated with external adaptive coping strategies or *Reappraisal*. In fact, *Reappraisal: Illness as Chance *correlated best with *Trust in Divine Help*, weakly with the other strategies, but not with *Trust in Medical Help*.

With respect to *Trust in Medical Help*, this strategy was moderately associated with *Search for Information/Alternative Help *which is plausible from a theoretical point of view, and consistently not with depressive *Escape from Illness*.

*Escape from Illness *(which is not regarded as an adaptive coping strategy) was associated weakly negative with life satisfaction and *Positive Attitudes*, but with none of the other adaptive strategies.

With respect to the interpretations of illness, particularly the positive interpretations (i.e., value and challenge) correlated weakly with adaptive coping strategies, but not with *Trust in Medical Help*. Among the interpretations with a negative connotation, none correlated with the adaptive coping strategies - there was just a weak positive association between *Trust in Medical Help *and adverse Interruption/Loss and a weak positive correlation between *Reappraisal *and Weakness/Failure (Table 3). Yet, all negative interpretations correlated moderately with *Escape from Illness *which is plausible.

Life Satisfaction was moderately associated particularly with active internal strategies *Conscious and Healthy Way of Living *and *Positive Attitudes*, while *Trust in Medical Help *and also the *Reappraisal *attitude did not correlate. Moreover, life satisfaction did not significantly correlate with the positive disease interpretation Value (r = -.055) or Challenge (r = .061).

Duration of disease did neither significantly correlate with the adaptive coping strategies nor with *Escape from Illness *(data not shown), and just marginally with life satisfaction (r = .201; p = .011).

To determine the relevance of the obvious intercorrelations between adaptive coping strategies on the one hand, and life satisfaction and positive interpretations of disease on the other hand, we performed regression analyses. The variables were enrolled on the basis of variance and correlation analyses. For this analysis, the positive interpretations of disease (i.e., Challenge and Value) were combined to a single factor.

As shown in Table 4, the standardized beta coefficients indicate that *Positive Attitudes *and age were positive predictors of life satisfaction, while C*onscious and Healthy Way of Living *(which was strongly correlated with *Positive Attitudes*) had just a marginal influence. Particularly *Reappraisal*, which was not among the relevant predictors of life satisfaction, exhibited some degree of collinearity. With respect to the positive interpretation of disease, *Reappraisal: Illness as Chance *had a significant influence, but none other adaptive coping strategies (Table 4).

**Table 4:** Influencing variables (regression model)

						Collinearity statistics	
						
Dependent Variables	Included variables	R^2^	Beta	T	**Sign**.	Toler	VIF
**Life Satisfaction**	(constant)	.331		1.240	.217		
	Trust in God's Help		.146	1.539	.126	.603	1.658
	Trust in Medical Help		-.032	-.386	.700	.810	1.234
	Search for Information/Alternative Help		-.064	-.741	.460	.725	1.379
	Positive Attitudes		**.417**	**4.511**	**.000**	.630	1.586
	Conscious Way of Living		.170	1.876	.063	.653	1.530
	Reappraisal: Illness as Chance		-.209	-1.854	.066	.425	2.355
	Escape from Illness		-.043	-.542	.589	.851	1.175
	Positive Interpretations of disease (Challenge/Value)		-.033	-.360	.720	.648	1.543
	Women		-.038	-.483	.630	.882	1.133
	Age		**.268**	**3.434**	**.001**	.888	1.126
	Educational level		.119	1.526	.129	.892	1.121
	Duration of disease		-.103	-1.316	.191	.878	1.139

**Positive Disease interpretation**	(constant)	.353		2.375	.019		
	Trust in God's Help		.019	.199	.843	.592	1.690
	Trust in Medical Help		-.067	-.834	.406	.814	1.229
	Search for Information/Alternative Help		.019	.219	.827	.722	1.385
	Positive Attitudes		-.001	-.007	.994	.542	1.846
	Conscious Way of Living		-.054	-.594	.553	.637	1.569
	Reappraisal: Illness as Chance		**.523**	**5.120**	**.000**	.500	1.998
	Escape from Illness		-.126	-1.619	.108	.867	1.153
	Life Satisfaction		-.032	-.360	.720	.669	1.494
	Women		.027	.335	.738	.812	1.232
	Age		-.150	-1.975	.051	.908	1.101
	Educational level		.075	.980	.329	.882	1.134
	Duration of disease		-.062	-.802	.424	.870	1.149

*Abbreviations*: Sign.- significance; Toler - Tolerance; VIF - variance inflation factor Variables with a significant influence were highlighted.

## Discussion

This study describes the use of adaptive coping styles referring to the concept of 'locus of disease control' among patients with chronic pain conditions. Most patients tended to externalize the process of disease management, i.e., the chronic pain disease was regarded as an adverse interruption of life, and thus patients called experts for help (i.e., medical doctors or therapists), and followed their advices or relied on the effects of prescribed remedies (see scale *Trust in Medical Help*) which is a rather passive strategy. The reliance on medical specialists to control or manage the problem of chronic pain nevertheless may go along with (internal) cognitive-behavioural changes, i.e., patients may change distinct aspects of their life, try to become more consciously, healthy, physically fit, use distinct diets etc. (see scale *Conscious and Healthy Way of Living*), or try to think positive, resolve cumbering situations of past, realize shelved dreams and wishes etc. (see scale *Positive Attitudes*); both are active strategies. However, in face of an insufficient manageability of chronic pain, some patients may call upon 'more powerful' external others (i.e., *Trust in Divine Help)*, because the conventional resources of help seem to be (subjectively) exhausted. Although the adaptive coping strategies may change during the individual course of disease, the mean scores did not significantly differ with respect to the duration of disease in the whole group. There were no steady-going courses of the adaptive coping strategies but transient trends with strong variances, indicating that the individual reasons to utilize a distinct strategy cannot be explained by duration of disease alone but by several influences (i.e., acceleration of pain intensity, treatment efficacy, partner support, depression, daily life management, financial situation, etc.).

In contrast to patients with cancer which have a strong reliance on external sources respectively *Trust in God's Help *[4,5], patients with chronic pain diseases had just a moderate utilization of this external resource [16]. To explain these differences in the utilization of intrinsic religiosity as an adaptive coping strategy between patients with cancer or chronic pain conditions, one may argue that cancer patients in general were much older, comprised a higher proportion of religious individuals, had a higher educational level - and a shorter course of disease [4,5,16,17]. In particular, the last argument might lead to the conclusion that cancer patients would rely more hopefully on spiritual sources than patients with chronic pain diseases which may have experienced the limitations of pain management, and may feel abandonment by higher sources during their suffering. As a result, they may have lower trust in God's help. Moreover, one may also suggest that cancer patients may be threatened by the possibility of death and thus have more inclination do deal with hope for God's help.

In terms of life satisfaction, particularly the internal coping strategy *Positive Attitudes *was the strongest predictor (along with age) and not *Conscious Way of Living *(although both were strongly in intercorrelated); all other adaptive coping strategies and also the positive interpretations of disease (i.e., Value and Challenge) did not significantly contribute to life satisfaction. This means that the cognitive behavioural strategy which relies on the intention of positive thinking, the avoidance of constantly thinking at illness, and the intention to take life in own hands, the realization of shelved dreams and wishes, the resolving of cumbering situations of the past and doing all that what pleases is of outstanding importance for patients with chronic diseases to cope. One might interpret this behaviour as patients' intention to leave the role model of a 'passive sufferer', and to become an active, self-actualizing individual.

Nevertheless, it is important to point out that even patients with impaired perception of health status can have high satisfaction with various dimensions of life concerns, i.e., they may have ground or find satisfaction in the relations with friends and families etc. While it is true that patients with chronic disease may experience decreased quality of life and life satisfaction, it is not necessarily true for all individuals. In a recent study we have shown that several dimensions of life satisfaction of patients with chronic pain diseases can score high despite of the experience of chronic pain [16].

According to Lipowski's original thesis, the experience of illness may enhance intensity and depth of life [13], and thus one could explain the association between internal adaptive coping strategies (particularly *Reappraisal*) and positive interpretations of disease. This means, patients have to find access to adequate resources - whatever these may be. Active adaptive coping strategies were not among the significant predictors of positive interpretations, but *Reappraisal *(which can be regarded as an active internal strategy to re-interpret illness and to find congruence with the impaired situation).

In contrast to cancer patients who regard their disease either as an interruption of life or even as a challenge, the patients with chronic pain conditions investigated herein predominantly regarded their disease as a loss. Consequently, patients' negative interpretations of disease were associated with *Escape from Illness*. Nevertheless, those who relied on a transcendent resource to cope (i.e., *Trust in Divine Help*) may regard their disease as a challenge or value. This unique view that illness could be a challenge or something of value can be found particularly in cancer patients [12,18,19].

A limitation of the paper is that we relied on data from a cross sectional study. The adaptive coping strategies may change during the course of disease, and patients have to adapt their strategies to changing situations. This has to be address in future longitudinal studies. It was striking that particularly patients from the acute pain outpatient clinic, which attended the clinic because of severe and acute pain episodes, had both the highest scores for *Escape from Illness *and the highest trust in external help (i.e., *Trust in Medical Help *and *Trust in Divine Help*) as compared to patients from the rehabilitation clinic or patients attending the mind-body program. Because we had no access to reliable data on patients' intensity of pain (which limits the interpretation of data), we can just assume that patients from the acute pain out-patient clinic had higher pain intensities as compared to the patients from the rehabilitation clinic and the mind-body training programs. In fact, they seemed to have had the strongest need for external intervention. Nevertheless, despite of their obvious needs, they retained the attitude to care for themselves (particularly, they had the highest scores for *Search for Information and Alternative Help*, and high scores for *Conscious and Healthy Living*) and to retain *Positive Attitudes *in life. Why the patients from the mind-body training program had lower score for the adaptive coping strategies remains to be clarified in longitudinal studies, because one may assume that - even if their attitudes are significantly different at the start of the program - the utilization of these strategies may change during the intervention program.

Nevertheless, to restore a sense of self-control over pain as well as the conviction that one is not necessarily disabled by disease and that pain is not necessarily a sign of damage [20] is a major task in patient care. Apart from effective pain management, a comprehensive approach is needed which enhances the psycho-spiritual well-being, i.e. self-awareness, coping and adjusting effectively with stress, relationships, sense of faith, sense of empowerment and confidence, and living with meaning and hope [21]. Also changing negative illness interpretations and depressive or avoidance coping by means of an intervention and encouraging social support by means of patient support groups may at least improve quality of life. Further studies are required, particularly longitudinal studies to measure changes in the weighting of adaptive coping strategies and interpretations of disease with respect to pain intensity, and comprehensive intervention programs.

## Conclusions

The experience of illness may enhance intensity and depth of life, and thus one may explain the association between internal adaptive coping strategies (particularly *Reappraisal*) and positive interpretations of disease. In the context of health services research, apart from effective pain management, a comprehensive approach is needed which enhances the psycho-spiritual well-being of patients with chronic pain diseases.

## Competing interests

The authors have no competing interests, and were free to interpret the data according to a strict scientific rationale. We disclose any funding received for this work from any organization.

## Authors' contributions

AB initiated the project, analysed and interpreted the data, and has written the manuscript. TO contributed to data analysis and the writing of the paper. EAMN and PH contributed to interpretation and the writing of the paper. All authors have read and approved the final manuscript.

## Pre-publication history

The pre-publication history for this paper can be accessed here:

http://www.biomedcentral.com/1471-2458/10/507/prepub

## References

[B1] Brown GK Nicassio PM Development of a questionnaire for the assessment of active and passive coping strategies in chronic pain patients Pain 1987 31 53 64 10.1016/0304-3959(87)90006-6 3696743

[B2] Ramirez-Maestre C Esteve R Lopez AE Cognitive appraisal and coping in chronic pain patients Eur J Pain 2008 12 749 756 10.1016/j.ejpain.2007.11.004 18096418

[B3] Ersek M Turner JA Kemp CA Use of the chronic pain coping inventory to assess older adults' pain coping strategies J Pain 2006 7 833 842 10.1016/j.jpain.2006.04.002 17074625

[B4] Büssing A Ostermann T Matthiessen PF Adaptive coping and spirituality as a resource in cancer patients Breast Care 2007 2 195 202 10.1159/000104172

[B5] Büssing A Ostermann T Matthiessen PF Wer kontrolliert die Gesundheit? - Adaptive Krankheitsverarbeitungsstile bei Patienten mit chronischen Erkrankungen Deutsche Zeitschrift für Onkologie 2008 40 150 156 10.1055/s-0028-1082647

[B6] Lipowski ZJ Psychosocial reactions to physical illness Can Med Assoc J 1983 128 1069 1072 6839255 PMC1874857

[B7] Büssing A Keller N Michalsen A Moebus S Ostermann T Matthiessen PF Spirituality and Adaptive Coping Styles in German Patients with Chronic Diseases in a CAM Health Care Setting Journal of Complementary and Integrative Medicine 2006 3 4 1 16 10.2202/1553-3840.1049

[B8] Rotter J Generalized expectations for internal versus external control reinforcement Psychological Monographs: General and Applied Psychology 1966 80 1 27 5340840

[B9] Tan G Nguyen Q Anderson KO Jensen M Thornby J Further validation of the chronic pain coping inventory J Pain 2005 6 29 40 10.1016/j.jpain.2004.09.006 15629416

[B10] Levenson H Distinctions Within the Concept of Internal-External Control: Development of a New Scale Proceedings of the 80th Annual Convention of the American Psychological Association 1972 7 261 262

[B11] Büssing A Matthiessen PF Mundle G Emotional and rational disease acceptance in patients with depression and alcohol addiction Health Qual Life Outcomes 2008 6 4 1 11 18208595 10.1186/1477-7525-6-4 PMC2262062

[B12] Büssing A Fischer J Interpretation of illness in cancer survivors is associated with health-related variables and adaptive coping styles BMC Women's Health 2009 2 1 11 19178733 10.1186/1472-6874-9-2 PMC2661070

[B13] Lipowski ZJ Physical illness, the individual and the coping processes Psychiatry Med 1970 1 91 102 4257952 10.2190/19q3-9ql8-xyv1-8xc2

[B14] Büssing A Fischer J Haller A Ostermann T Matthiessen PF Validation of the Brief Multidimensional Life Satisfaction Scale in patients with chronic diseases Eur J Med Res 2009 14 171 177 19380290 10.1186/2047-783X-14-4-171 PMC3401007

[B15] Munce SE Stewart DE Gender differences in depression and chronic pain conditions in a national epidemiologic survey Psychosomatics 2007 48 394 399 10.1176/appi.psy.48.5.394 17878497

[B16] Büssing A Michalsen A Balzat HJ Grunther RA Ostermann T Neugebauer EA Matthiessen PF Are spirituality and religiosity resources for patients with chronic pain conditions? Pain Med 2009 10 327 339 10.1111/j.1526-4637.2009.00572.x 19284487

[B17] Büssing A Ostermann T Koenig HG Relevance of religion and spirituality in German patients with chronic diseases Int J Psychiatry Med 2007 37 39 57 10.2190/60W7-1661-2623-6042 17645197

[B18] Degner LF Hack T O'Neil J Kristjanson LJ A new approach to eliciting meaning in the context of breast cancer Cancer Nurs 2003 26 169 178 10.1097/00002820-200306000-00001 12832949

[B19] Wallberg B Michelson H Nystedt M Bolund C Degner L Wilking N The meaning of breast cancer Acta Oncol 2003 42 30 35 10.1080/0891060310002203 12665328

[B20] Nielson WR Jensen MP Relationship between changes in coping and treatment outcome in patients with Fibromyalgia Syndrome Pain 2004 109 233 241 10.1016/j.pain.2004.01.002 15157683

[B21] Lin HR Bauer-Wu SM Psycho-spiritual well-being in patients with advanced cancer: an integrative review of the literature 2003 44 69 80 12956671 10.1046/j.1365-2648.2003.02768.x

